# Spatial Analysis of Dengue Seroprevalence and Modeling of Transmission Risk Factors in a Dengue Hyperendemic City of Venezuela

**DOI:** 10.1371/journal.pntd.0005317

**Published:** 2017-01-23

**Authors:** Maria F. Vincenti-Gonzalez, María-Eugenia Grillet, Zoraida I. Velasco-Salas, Erley F. Lizarazo, Manuel A. Amarista, Gloria M. Sierra, Guillermo Comach, Adriana Tami

**Affiliations:** 1 Department of Medical Microbiology, University of Groningen, University Medical Center Groningen, Groningen, The Netherlands; 2 Laboratorio de Biología de Vectores y Parásitos, Instituto de Zoología y Ecología Tropical, Facultad de Ciencias, Universidad Central de Venezuela, Caracas, Venezuela; 3 Centro de Estudio de Enfermedades Endémicas y Salud Ambiental, Instituto de Altos Estudios “Dr. Arnoldo Gabaldón”, Ministerio de Salud, Maracay, Aragua, Venezuela; 4 Instituto de Investigaciones Biomédicas “Dr. Francisco J. Triana-Alonso”, Universidad de Carabobo, Maracay, Venezuela; 5 Departamento de Parasitología, Facultad de Ciencias de la Salud, Universidad de Carabobo, Valencia, Venezuela; University of California, Davis, UNITED STATES

## Abstract

**Background:**

Dengue virus (DENV) transmission is spatially heterogeneous. Hence, to stratify dengue prevalence in space may be an efficacious strategy to target surveillance and control efforts in a cost-effective manner particularly in Venezuela where dengue is hyperendemic and public health resources are scarce. Here, we determine hot spots of dengue seroprevalence and the risk factors associated with these clusters using local spatial statistics and a regression modeling approach.

**Methodology/Principal Findings:**

From August 2010 to January 2011, a community-based cross-sectional study of 2012 individuals in 840 households was performed in high incidence neighborhoods of a dengue hyperendemic city in Venezuela. Local spatial statistics conducted at household- and block-level identified clusters of recent dengue seroprevalence (39 hot spot households and 9 hot spot blocks) in all neighborhoods. However, no clusters were found for past dengue seroprevalence. Clustering of infection was detected at a very small scale (20-110m) suggesting a high disease focal aggregation. Factors associated with living in a hot spot household were occupation (being a domestic worker/housewife (*P* = 0.002), lower socio-economic status (living in a shack (*P*<0.001), sharing a household with <7 people (*P* = 0.004), promoting potential vector breeding sites (storing water in containers (*P* = 0.024), having litter outdoors (*P* = 0.002) and mosquito preventive measures (such as using repellent, *P* = 0.011). Similarly, low socio-economic status (living in crowded conditions, *P*<0.001), having an occupation of domestic worker/housewife (*P* = 0.012) and not using certain preventive measures against mosquitoes (*P*<0.05) were directly associated with living in a hot spot block.

**Conclusions/Significance:**

Our findings contribute to a better comprehension of the spatial dynamics of dengue by assessing the relationship between disease clusters and their risk factors. These results can inform health authorities in the design of surveillance and control activities. Focalizing dengue control measures during epidemic and inter-epidemic periods to disease high risk zones at household and neighborhood-level may significantly reduce virus transmission in comparison to random interventions.

## Author Summary

Dengue is a mosquito-borne viral disease of global impact. In Venezuela, dengue is endemic with the co-circulation of the 4 viral serotypes and has become one of the most important public health problems of urban areas. During 2010 and 2011, a baseline cross-sectional study was carried out as part of a dengue prospective cohort study. We enrolled 2012 individuals aged 5–30 years living in 840 households within 3 neighbourhoods in Maracay, one of the cities with the highest number of reported dengue cases in Venezuela. Serological data were obtained through blood sample collections prior to informed consent. Analyses of risk-maps at a fine scale were carried out to detect dengue seroprevalence hot spots (areas of greater transmission) within these neighborhoods and to relate them with potential transmission risk factors. Recent dengue infection clustered within and around households and blocks (radius 20-110m), suggesting the relevant spatial scale at which disease transmission occurs in the studied area. People that lived within these hot spots were poorer and had more man-made potential mosquito breeding sites in and around their premises. Focalizing dengue control measures to infection high risk zones may result in a more cost effective approach of dengue surveillance and control. Spatial statistics analyses are powerful tools to identify the past and the actual distribution of dengue, localize high risk areas and help focalizing control measures.

## Introduction

The incidence of dengue, a vector-borne viral disease, has risen markedly in the last decades affecting more than half of the world’s population [1]. According to a recent study, 390 million dengue infections are estimated to occur annually [2]. Dengue and its vectors have spread into previously unaffected areas and presently this disease is endemic in 128 countries [3,4]. Currently, dengue control methods rely mostly on vector reduction; however, these activities have proven largely unsuccessful [4].

Geographic information systems (GIS) and spatial analysis techniques are important tools for public health as they integrate the detection of disease spatial patterns, the identification of unusual aggregations (hot spots) of epidemiological events and allow the prediction of high risk areas of disease transmission [5,6]. Dengue hot spots identification is suitable to focalize health control measures and epidemiological surveillance in a cost effective manner particularly in regions where resources are limited [7,8].

Dengue virus (DENV) belongs to the *Flavivirus* genus of the family *Flaviviridae* [9]. It is transmitted by the bite of infected female *Aedes* mosquitoes, mainly *Ae*. *aegypti* [10]. Although *Ae*. *albopictus* is a less efficient vector; it has also been related to dengue outbreaks [11]. DENV consists of four serologically distinct serotypes (DENV-1 to -4) each of them capable of causing the entire range of dengue-related disease symptoms [12].

In Venezuela, dengue has become a major public health problem of urban areas. DENV transmission is endemic with the co-circulation of the 4 viral serotypes [13]. Control of this infection and of its mosquito vector has proven challenging due to growing population density, increasingly crowded living conditions, unreliable water supply, and enduring problems in public services [14,15,16]. Furthermore, in recent years Venezuela experienced an increase in dengue incidence; with this increase being related to health sector crisis, budget cuts and shortage of medicines due to technical and economical limitations [16,17,18]. Despite control measures, transmission of dengue in Venezuela has become persistent with an average of 40,000 cases annually in non-epidemic years and three large epidemics in the past decade [19]. The most recent and biggest occurred in 2010, where approximately 125,000 cases including 10,300 (8.6%) with severe manifestations were registered [19]. Maracay city in Aragua state has become one of the most important endemic urban areas in the country. The highest number of cases and dengue incidence during the 2010 epidemic was reported in Aragua (495 cases per 100.000 inhabitants) [14,19]. Indeed, during this year, Venezuela was the country with the third highest number of reported dengue cases in the Americas and ranked second in the number of severe cases [20]. National dengue control measures involve strategies to reduce the vector [21,22] however, in recent years surveillance and control measures have been applied irregularly or have been absent [17,18].

Previous studies in Maracay, a dengue hyperendemic city in Venezuela, have shown that certain areas are more prone to maintain higher dengue transmission and for longer periods than others [15] indicating that some epidemiological conditions are stable through time. Using mapping technology and spatial analysis of epidemiological and seroprevalence data we attempt to draw risk-maps at a fine scale to identify clusters (hot spots) of DENV transmission within high dengue incidence neighborhoods in Maracay and relate them with the risk factors present in the studied areas. Results will be used to inform health authorities to improve dengue control strategies.

## Methods

### Area of Study

**Fig 1:**
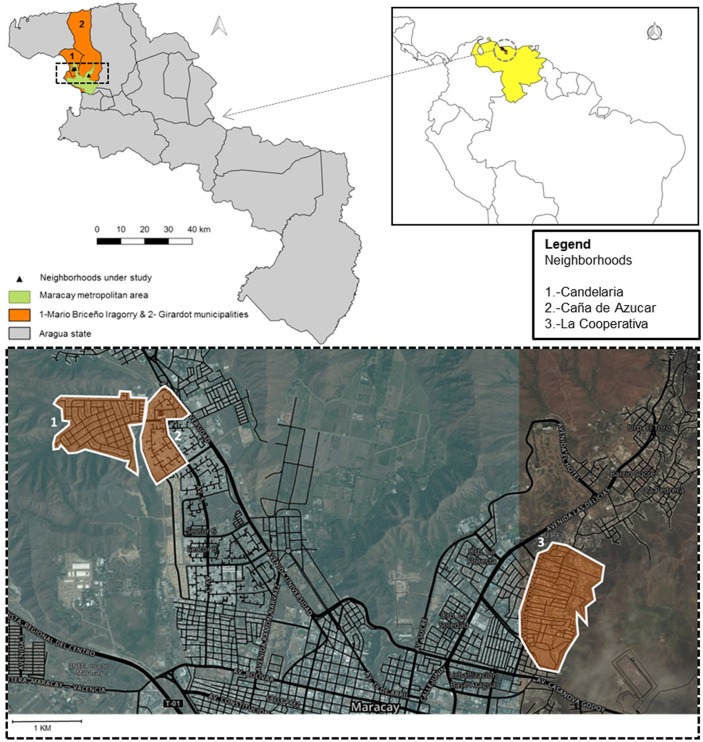
Study area. Location of the three study sites: 1) Candelaria 2) Caña de Azucar and 3) La Cooperativa neighborhoods within the metropolitan area of Maracay city, Aragua State, Venezuela.

**Fig 2:**
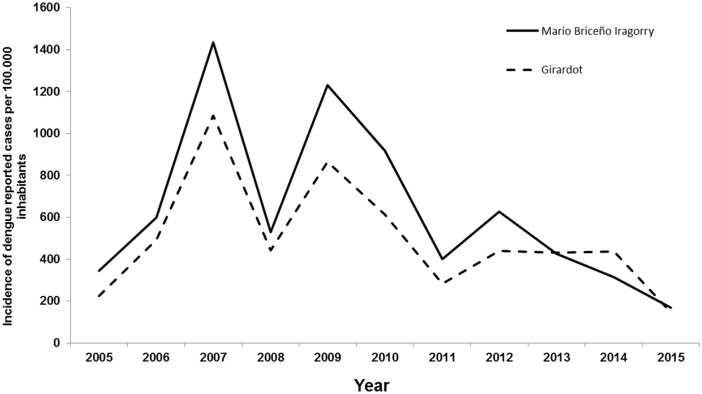
Dengue incidence in Mario Briceño Iragorry and Girardot municipalities, Aragua state, between 2005–2015. Mario Briceño Iragorry municipality contains the neighborhoods of Caña de Azucar and Candelaria, while La Cooperativa neighborhood lies within Girardot municipality. Source: LARDIDEV (Laboratorio Regional para el Diagnóstico del Dengue y otras Enfermedades Virales), Corporación de Salud del Estado Aragua (CORPOSALUD Aragua), Ministerio del Poder Popular para la Salud, Venezuela, 2016.

Maracay is the capital city of Aragua state in the north-central region of Venezuela (10° 15′ 6″ N, 67° 36′ 5″ W) with an estimated population of 1.139.000 inhabitants [23]. The annual average temperature is 25.5°C (min 19°C, max 31°C) with 74% of humidity and an annual precipitation of 910 mm [24] with two seasons, a dry (November-April) and a rainy season (May-October). Three neighborhoods within two municipalities of high dengue incidence [15] were chosen for our study (Fig 1). The reported dengue incidence is slightly higher in Mario Briceño Iragorry municipality than in Girardot municipality (Fig 2). Lately, two dengue transmission peaks took place in these municipalities; one in 2007 and the second in 2009–2010 parallel to the whole dengue incidence in Aragua State and Venezuela as shown previously [14]. Caña de Azúcar and Candelaria neighborhoods, belonging to Mario Briceño Iragorry municipality, are located close to each other in the north-western area of Maracay. This municipality has a population of 99,852 inhabitants in an area of 54 km^2^ [23]. Caña de Azúcar and Candelaria neighborhoods have an area of 0,50 and 0,87 km^2^, respectively; are divided by the “Limón” river and surrounded northerly by the mountainous National park “Henri Pittier” (Fig 1). Cooperativa neighborhood (Girardot municipality) is found in the north-east side of Maracay with an area of 1.1 km^2^ and the river “Las Delicias” running along its eastern border (Fig 1) [25]. Girardot municipality has a population of 590,679 inhabitants in an area of 312 km^2^ [23]. Both municipalities are located within the metropolitan area of Maracay which comprises pre-planned urban areas. However, unplanned developments are also present and are characterized by the lack of public services such as electricity, water supply and garbage collection. In addition, piped-water supply is irregular in the entire Aragua state compelling the population to store water in tanks and other containers in order to ensure constant access to water [14,15].

### Study Design

A baseline cross-sectional study was carried out during the recruitment process of a prospective community-based cohort study described elsewhere [14]. Briefly, 2012 individuals aged 5–30 years inhabiting 840 households within the above mentioned neighborhoods were enrolled, from August 2010 to January 2011, through house-to-house visits. The inclusion criteria were described previously [14], briefly: age between 5–30 years old; living in the study area with no intention to move in the following 3 years; and consenting to attend the designated health centre in case of any symptoms. The scope of the study was clearly explained to all members of the household and the individuals were enrolled after written informed consent. Data were collected through an individual and a household structured questionnaires. The individual questionnaire contained data related to socio-demographic, epidemiologic and clinical history while socio-economic and environmental factors were recorded with the household questionnaire. Serological and hematological data were acquired through blood sample collections [14]. The geographical location of each household was obtained using a hand-held Global Positioning System (GPS, Garmin Ltd.). As reported earlier [14], 5–10% of households with a probable higher socio-economic status in Cooperativa refused to participate. Since socio-economic variables were similar between Cooperativa and Candelaria where refusal was minimal, we believe that selection bias in Cooperativa was small. In the present study, 1985 individuals living in 837 households that had a recorded geographical position were included. They were distributed as follows: Candelaria with 452 individuals living in 208 households, Cooperativa with 601 people in 266 households and Caña de Azucar where 932 subjects inhabited 363 residences.

### Dengue Seroprevalence

A 10 ml blood sample was collected from each enrolled individual to perform baseline dengue serology and a full blood count. Dengue seropositivity was determined using the Hemaglutination Inhibition (HI) Assay as described in detail in Velasco *et al*.,[14]. Two variables for previous dengue infection were defined: a) Past dengue infection: HI titres >1:20, and b) Recent dengue infection: HI titres ≥ 1280 [14,26]. As reported earlier, 77.4% of the population under study had a past dengue infection while 10% exhibited a recent infection. The latter was more prevalent in Caña de Azucar (12.8%), followed by Cooperativa (8.1%) and Candelaria (7.4%) neighborhoods [14].

### Spatial Analysis: Hot Spot Detection

The hypothesis that an event of dengue infection is equally likely to occur at any location within the study area, regardless of the locations of other events, was tested. For that, we used one local measure of spatial association, the local Getis statistic [27]. The event was analyzed at two spatial scales: 1) at household level, and 2) at block level. Dengue seroprevalence at block and household level was standardized as the total number of seropositive individuals divided by the total number of individuals surveyed in a block or household, respectively. Risk maps at block and household level were drawn for the two seroprevalence outcome variables. The local Getis statistic, *G*i*(*d*) detected significant local clustering of high positive (hot spots) values of dengue prevalence around each point (e.g. household infection) within a radius (circular window) of specified distance *d* from that location. The distance *d* defined the neighborhood search for a particular house or block, with nearby locations being expected to have similar values. The value obtained was compared (by using the Monte Carlo randomization procedure) with the statistic’s expected value to indicate if the degree of clustering of dengue prevalence in the vicinity of a particular location was greater or less than expected by chance. To correct for multiple comparisons when using *G*i*(*d*), significance levels (*P* < 0.05) were adjusted according to Ord & Getis (1995) [28]. We calculated *G*i*(*d*) at different window sizes with the maximum *G*i*(*d*) distance corresponding to the scale at which the *G*i*(*d*) maximum value was found that is, the scale of the spatial dependence of the process under study [29]. The analyses of *G*i*(*d*) were carried out through the Point Pattern Analysis (PPA 1.0, San Diego State University, San Diego, CA http://www.acsu.buffalo.edu/~geojared/tools.htm). The results were shown in maps using the softwares QGIS 1.8.0-Lisboa (GNU—General Public License) and ArcGIS 10 (ESRI Corporation, Redlands, CA). The satellite images of each neighborhood were obtained from Google EarthTM.

### Univariate and Multivariate Risk Factor Analysis

Two outcome variables were defined based on the Getis analysis of recent dengue seroprevalence hot spot detection: 1) Individuals living in a hot spot household, and 2) Individuals living in a hot spot block. Univariate and multivariate analyses of potential risk factors associated to hot spots at household and block level of recent dengue infection were performed using SPSS (SPSS Inc., version 20.0, Chicago, Illinois) software. Variables included in the analysis were the following: demographic (age, gender, occupation), socio-economic (duration of residence, studying and having a job, type of housing, number of household rooms, persons per household), crowding (number of persons living in a household divided by the number of household rooms), environmental (water storage at home, availability of public services, presence of litter, used car tires and bottles outdoor and indoor flower vases), and mosquito preventive measures (screened windows/doors, use of mosquito nets, insecticide and repellent usage, and container washing). The studied variables were previously described in detail [14]. Variables considered as confounders were age and gender. Continuous variables were converted into ordered categorical variables when suitable; otherwise they were dichotomized above and below their mean value (if normally distributed) or the median (when non-normally distributed). The variable crowding was divided into quartiles and a cut-off point was set between the third and fourth quartiles (~1.5persons/room) where a difference in prevalence was observed. Proportions were compared using chi-square tests. Fisher’s exact test was used when one or more cells of the contingency table had an expected count of less than five. Logistic regression was used to compare crude and adjusted odds ratios (OR). Significance was determined at the 5% level (*P* < 0.05). The final models contained variables independently associated with living in a hot spot household or hot spot block.

### Ethics Statements

Data were analyzed anonymously and individuals were coded with unique numeric identifiers. All adult subjects ≥18 years old provided written informed consent, and a parent or guardian of any child participant provided written informed consent on their behalf. Children between 8 and 17 years old provided written informed assent [14]. The study was approved by the Ethics Review Committee of the Biomedical Research Institute, Carabobo University (Aval Bioetico #CBIIB(UC)-014), Maracay, Venezuela; the Ethics, Bioethics and Biodiversity Committee (CEBioBio) of the National Foundation for Science, Technology and Innovation (FONACIT) of the Ministry of Science, Technology and Innovation, Caracas, Venezuela; and by the Regional Health authorities of Aragua State (CORPOSALUD Aragua). The study was conducted according to the principles expressed in the Declaration of Helsinki [30].

## Results

### Risk Maps and Hot Spot Detection

**Fig 3:**
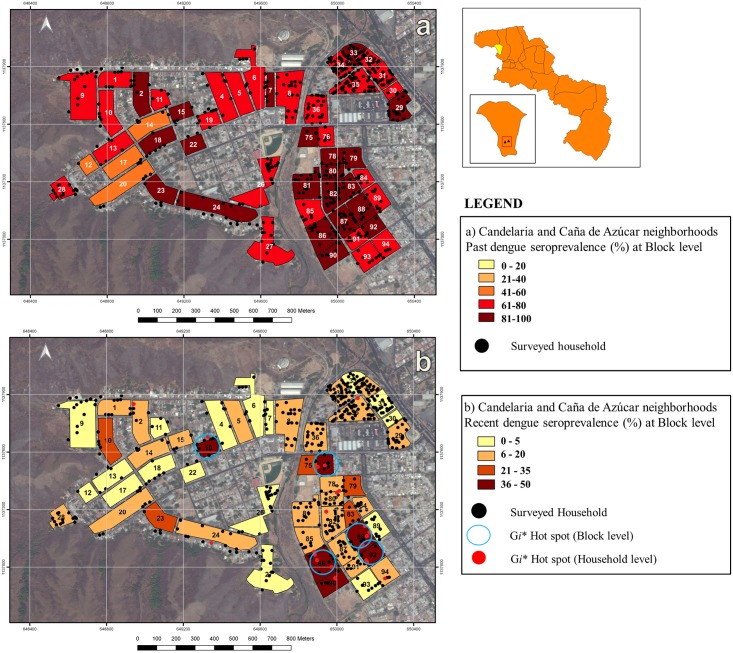
Dengue seroprevalence within Candelaria & Caña de Azúcar neighborhoods, Maracay city, Venezuela. **a) Past dengue seroprevalence:** Blocks show past dengue seroprevalence (%). No hot spots at any spatial scale were found (*G*i*(*d*) < 2.79, *P* >0.05). Black dots indicate surveyed households. **b) Recent dengue seroprevalence:** Blocks show recent dengue seroprevalence (%). Blue encircled blocks show the results of the local Getis statistic (Gi*(*d*)) analyses at a distance of 90 meters, with significant (*P* <0.05) clusters of recent dengue infection at block level (hot spots). Red dots show the results of Gi*(*d*) analyses at a distance of 20 meters with significant clustering of recent dengue infection at household level (*G*i*(*d*) >2.79, *P* <0.05). Black dots indicate surveyed households.

**Fig 4:**
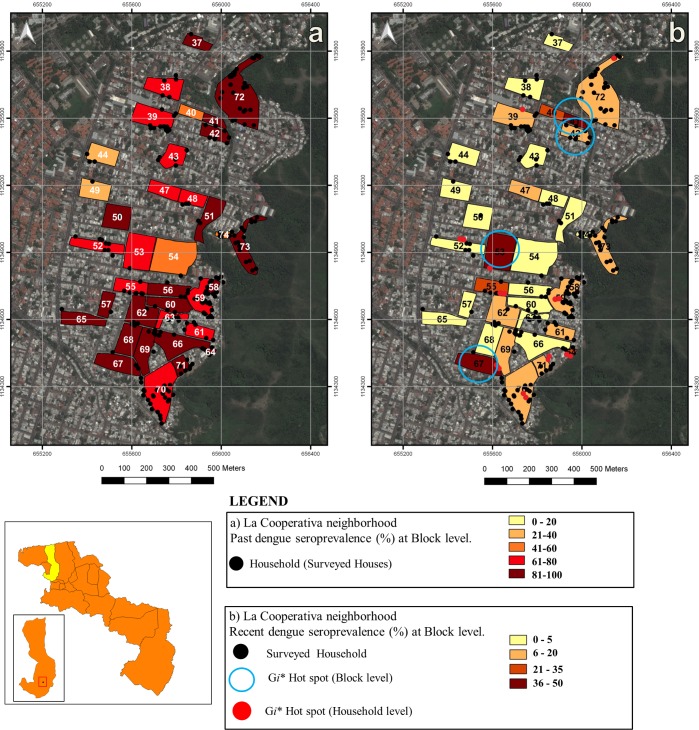
Dengue seroprevalence within La Cooperativa neighborhood, Maracay city, Venezuela. **a) Past dengue seroprevalence:** Blocks show past dengue seroprevalence (%). No hot spots at any spatial scale were found (*G*i*(*d*) < 2.79, *P* >0.05). Black dots indicate surveyed households. **b) Recent dengue seroprevalence:** Blocks show recent dengue seroprevalence (%). Blue encircled blocks show the results of the local Getis statistic (Gi*(*d*)) analyses at a distance of 110 meters, with significant (*P* <0.05) clusters of recent dengue infection at block level (hot spots). Red dots show the results of Gi*(d) analyses at a distance of 20 meters with significant clustering of recent dengue infection at household level (Gi*(*d*) >2.79, *P* <0.05). Black dots indicate surveyed households.

Risk maps of the spatial distribution and local clustering of dengue seroprevalence for each outcome variable (past and recent) at block and household level are shown in Figs 3 and 4. A high seroprevalence of past dengue infection was found across all neighborhoods, resulting in risk maps where most blocks exhibited a seroprevalence >40%. Consequently, no past dengue seroprevalence clusters were detected by the local spatial statistics at any spatial scale (household or block; Figs 3a and 4a). Risk maps of recent dengue seroprevalence depicted a greater spatial heterogeneity. The highest frequency of recent dengue transmission at block level (seroprevalence >36%) was located in the southern part of Caña de Azucar neighborhood, while the majority of Candelaria’s blocks and the rest of Caña de Azucar displayed a seroprevalence below 21% (Fig 3b). Most blocks within La Cooperativa neighborhood showed low seroprevalence except for three areas, one in the northern, the second one in the middle and the third one in the southern edge where a prevalence higher than 36% was found (Fig 4b). Significant hot spots at household and block level were identified for recent dengue seroprevalence. Spatial statistics showed that most of the recently infected individuals were spatially located toward the southern side of Caña de Azucar neighborhood (Fig 3b). The local Getis statistic identified in this southern area 3 hot spots at block level (blocks 86, 88 and 92) and 9 hot spots at household level (see below and Fig 3b). Consequently, the most relevant recent dengue transmission gathering of clusters was found in this particular neighborhood. Additionally, hot spots at household and block level were detected by the local Getis analysis in all neighborhoods with a total of 65 individuals residing in the 39 detected hot spot households while 144 people lived in 62 households within 9 hot spot blocks. Four clusters at block level were identified in Caña de Azúcar, one in Candelaria and four in La Cooperativa neighbourhood (Figs 3b and 4b). The four hot spots at block level found in Caña de Azucar contained 36 houses, 13 (36%) of which were also hot spots households. The cluster found in Candelaria contained 9 households and one (11%) hot spot household. In La Cooperativa neighborhood, four hot spots blocks were identified including 17 households of which 4 (23.5%) were hot spots. Finally, the spatial-scale (maximum Gi*(*d*) distance) at which all dengue clusters were detected varied between 20 meters for household level clusters and 90–110 meters for block level clusters suggesting the relevant spatial scale at which dengue transmission occurs in the studied urban landscape.

### Risk Factors Associated with Living in a Dengue Hot Spot

The values of the two outcome variables of recent dengue seroprevalence hot spot detection resulted as follows: Sixty five individuals living in hot spot households (n = 65/1985 = 3.3%), and 144 individuals living in hot spot blocks (n = 144/1985 = 7.3%).

#### Demographic risk factors

Univariate analysis of demographic risk factors for clustered households and blocks identified by local Getis analyses showed that age and gender were not significantly associated with living in a hot spot household or block (Table 1). Being a domestic worker/housewife was the only occupation category significantly associated with living in a hot spot household (OR = 5.08, *P* = 0.034). This association became stronger and also applied to individuals living within a hot spot block, when occupation was treated as a binary variable. That is, there was a positive association between being a domestic worker and living in a hot spot household (OR = 2.85, *P* < 005) or block (OR = 1.67, *P* < 0.05) compared to people having other jobs (Table 1)

**Table 1:** Univariate analysis of demographic risk factors associated with living in hot spot households and blocks.

		Hot spot households				Hot spot blocks			
	Total number of subjects	n (%)	Crude OR	CI_95_	*P*-value	n (%)	Crude OR	CI_95_	*P*-value
**Age group (years) (n = 1985)**									
5–10	449	13 (2.9)	1	-	-	32 (7.1)	1	-	-
11–15	406	11 (2.7)	0.93	0.41–2.10	0.869	32 (7.9)	1.12	0.67–1.85	0.675
16–20	390	13 (3.3)	1.16	0.53–2.53	0.715	33 (8.5)	1.21	0.73–2.00	0.471
21–30	740	28 (3.8)	1.32	0.67–2.57	0.417	47 (6.4)	0.88	0.56–1.40	0.603
**Gender (n = 1985)**									
M	852	26 (3.1)	1	-	-	60 (7.0)	1	-	-
F	1133	39 (3.4)	1.13	0.69–1.88	0.721	84 (7.4)	1.06	0.75–1.50	0.819
**Occupation (n = 1984)**									
Unemployed	124	2 (1.6)	1	-	-	9 (7.3)	1	-	-
Student	1363	41 (3.0)	1.90	0.45–7.91	0.383	97 (7.1)	0.98	0.48–1.99	0.953
Housewife/ Domestic worker	182	14 (7.7)	5.08	1.13–22.77	0.034	20 (11.0)	1.57	0.70–3.60	0.277
Manual worker	174	2 (1.1)	0.71	0.09–5.10	0.733	11 (6.3)	0.86	0.35–2.15	0.750
Merchant/ Employee/ Office worker	118	6 (5.1)	3.27	0.64–16.52	0.152	5 (4.2)	0.56	0.18–1.74	0.320
Professional/ University staff	22	0 (0.0)	0.00	0.00	0.998	2 (9.1)	1.28	0.26–6.35	0.765
**Domestic worker/housewife binary (n = 1983)**									
No	1801	51 (2.8)	1	-	-	124 (6.9)	1	-	-
Yes	182	14 (7.7)	2.85	1.55–5.27	0.001	20 (11.0)	1.67	1.01–2.75	0.060

#### Socio-economic risk factors

The stronger factor associated with living in hot spot households was residing in a shack/“Rancho” (OR = 13.51, *P* < 0.05), however the small sample of individuals living in this type of dwelling determines the wide OR confidence interval (Table 2). Unexpectedly, the odds of living in a hot spot household were negatively associated with a higher number of inhabitants (>6) per household (OR = 0.42, *P* = 0.004, Table 2). The number of household rooms, crowding (number of people per room), the time that people lived in the same residence, studying, having a job or not doing any of those at the time of the interview were not significantly associated with the odds of being in a hot spot household. Contrariwise, the odds of living in a hot spot block were positively associated with individuals residing in a household with more than six people (OR = 1.97, *P* <0.001) and in crowded conditions (OR = 2.32, *P* <0.001) but, negatively associated with those inhabiting a household with more than five rooms (OR = 0.54, *P* = 0.001). There was no association with the rest of the socio-economic factors (Table 2).

**Table 2:** Univariate analysis of socio-economic risk factors associated with living in hot spot households and blocks.

		Hot spot households				Hot spot blocks			
	Total number of subjects	n (%)	Crude OR	CI_95_	P-value	n (%)	Crude OR	CI_95_	P-value
**Type of housing (n = 1985)**									
House/Apartment/ other	1961	58 (3.0)	1	-	-	144 (7.3)	-	-	-
Rancho ^a^ (shack)	24	7 (29.2)	13.51	5.39–33.83	<0.001*	0 (0.0)	-	-	0.413*
**Number of household rooms (n = 1985)**									
1–4	536	24 (4.5)	1	-	-	57 (10.6)	1	-	-
≥5	1449	41 (2.8)	0.62	0.37–1.04	0.091	87 (6.0)	0.54	0.38–0.76	0.001
**Number of persons per household (n = 1985)**									
1–6	1165	50 (4.3)	1	-	-	62 (5.3)	1	-	-
≥7	820	15 (1.8)	0.42	0.23–0.75	0.004	82 (10.0)	1.98	1.04–2.79	<0.001
**Crowding (n = 1985) (N° people/room)**									
0.17–1.49	1381	43 (3.1)	1	-	-	74 (5.4)	1	-	-
1.5–8.0	604	22 (3.6)	1.18	0.70–1.98	0.637	70 (11.6)	2.32	1.64–3.26	<0.001
**Duration of residence (n = 1931)**									
0–16	1459	44 (3.0)	1	-	-	106 (7.3)	1	-	-
≥17	472	18 (3.8)	1.28	0.73–2.23	0.481	35 (7.4)	1.02	0.68–1.52	0.994
**People who did not study nor worked (n = 1985)**									
No	1691	51 (3.0)	1	-	-	119 (7.0)	1	-	-
Yes	294	14 (4.8)	1.61	0.88–2.94	0.169	25 (8.5)	1.23	0.78–1.93	0.440
**Following any type of study (n = 1982)**									
No	608	23 (3.8)	1	-	-	45 (7.4)	1	-	-
Yes	1374	42 (3.1)	0.80	0.48–1.35	0.484	99 (7.2)	0.97	0.67–1.40	0.951
**Having a job (n = 1982)**									
No	1521	53 (3.5)	1	-	-	116 (7.6)	1	-	-
Yes	461	12 (2.6)	0.74	0.39–1.40	0.434	28 (6.1)	0.78	0.51–1.20	0.306

^a^In Venezuela, the word “rancho” is used to define a “shack”, an informal substandard type of housing typical of slum areas.

(*)Fisher´s exact test

#### Household and environmental risk factors and mosquito preventive measures

The majority of individuals inhabiting hot spot houses (56/57 = 98%) stored water at home in general (in a tank and/or containers, Table 3). Storing water in containers was positively associated with living in a hot spot household (OR = 1.96, *P* = 0.031). People who lived in households where litter was found outdoors (garden or patio) had odds of residing in a hot spot household that were 2.37 times as high as those who did not have litter outdoors; while other common mosquito breeding sites such as used car tires outdoors, bottles outdoors and indoor flower vases did not show a statistically significant association. The availability of public services did not influence the odds of living in a hot spot household. Amongst the mosquito preventive measures (Table 4), people using personal mosquito repellents were positively and significantly associated (*P* = 0.013) with inhabiting hot spot households than those that did not use them. These individuals referred as reasons to use repellents the high amount of dengue cases and mosquitoes in their area of residence and surroundings. Other protective measures such as screened windows/doors, the use of insecticide sprays and water container washing were not associated with living in a hot spot household. Finally, using mosquito nets showed a protective effect (OR = 0.63) but given that only one person referred to use it, the relation was not statistically significant. Three variables were significantly associated with living within a hot spot block and those were mosquito preventive measures. The odds of individuals that used insecticide (OR = 0.44, *P* <0.001) and that washed their water containers regularly (OR = 0.39, *P* <0.001) were negatively associated with living in a hot spot block, while the odds of individuals that had screened windows/doors were positively associated with living in a hot spot block (OR = 2.14, *P* = 0.047) (Table 4). To store water in tanks is a common practice in the studied neighborhoods, due to the irregular piped water service, and the odds of people that store water in tanks were positively but not significantly associated with living in a hot spot block. Moreover, the presence of used car tires outdoors may create a suitable environment to develop mosquito breeding sites. However, the odds of people having used tires outdoors were positively but not significantly associated with living in a hot spot block (*P* = 0.105) (Table 3).

**Table 3:** Univariate analysis of household and environmental risk factors associated with living in hot spot households and blocks.

		Hot spot households				Hot spot blocks			
	Total number of subjects	n (%)	Crude OR	CI_95_	P-value	n (%)	Crude OR	CI_95_	P-value
**General water storage at home (n = 1977)**									
No	176	1 (0.6)	-	-	-	11 (6.3)	-	-	-
Yes	1801	56 (3.1)	5.62	0.77–40.89	0.092	125 (6.9)	1.12	0.59–2.12	0.850
**Water storage in tanks (n = 1977)**									
No	429	10 (2.3)	1	-	-	22 (5.1)	1	-	-
Yes	1548	47 (3.0)	1.31	0.66–2.62	0.542	114 (7.4)	1.47	0.92–2.35	0.131
**Water storage in containers (n = 1977)**									
No	848	16 (1.90)	1	-	-	66 (7.8)	1	-	-
Yes	1129	41 (3.6)	1.96	1.10–3.52	0.031	70 (6.2)	0.78	0.55–1.11	0.198
**Public services (n = 1985)**									
No	96	4 (4.2)	1	-	-	6 (6.3)	1	-	-
Yes	1889	61 (3.2)	0.77	0.27–2.16	0.553*	138 (7.3)	1.18	0.50–2.75	0.851
**Potential mosquito breeding sites:**									
**Litter outdoors (n = 1977)**									
No	1170	22 (1.9)	1	-	-	87 (7.4)	1	-	-
Yes	807	35 (4.3)	2.37	1.38–4.06	0.002	49 (6.1)	0.81	0.56–1.16	0.277
**Used car tires outdoors (n = 1977)**									
No	1774	49 (2.8)	1	-	-	116 (6.5)	1	-	-
Yes	203	8 (3.9)	1.44	0.67–3.09	0.466	20 (9.9)	1.56	0.95–2.57	0.105
**Bottles outdoors (n = 1977)**									
No	1276	33 (2.6)	1	-	-	95 (7.4)	1	-	-
Yes	701	24 (3.4)	1.34	0.78–2.27	0.355	41 (5.8)	0.77	0.53–1.13	0.212
**Indoor flower vases (n = 1977)**									
No	882	24 (2.7)	1	-	-	59 (6.7)	1	-	-
Yes	1095	33 (3.0)	1.11	0.65–1.89	0.802	77 (7.0)	1.06	0.74–1.50	0.834

(*)Fisher´s exact test.

**Table 4:** Univariate analysis of mosquito preventive measures associated with hot spot households and blocks.

		Hot spot households				Hot spot blocks			
	Total number subjects	n (%)	Crude OR	CI_95_	P-value	n (%)	Crude OR	CI_95_	P-value
**Screened Windows/Doors (n = 1977)**									
No	1909	55 (2.9)	1	-		127 (6.7)	1	-	
Yes	68	2 (2.9)	1.02	0.24–4.27	1.000*	9 (13.2)	2.14	1.04–4.42	0.047*
**Mosquito net (n = 1969)**									
No	1915	56 (2.9)	1	-	-	129 (6.7)	1	-	-
Yes	54	1 (1.9)	0.63	0.09–4.61	1.000*	7 (13.0)	2.06	0.91–4.65	0.094*
**Insecticide (n = 1977)**									
No	985	26 (2.6)	1	-	-	93 (9.4)	1	-	-
Yes	992	31 (3.1)	1.19	0.70–2.02	0.610	43 (4.3)	0.44	0.30–0.63	<0.001
**Repellent (n = 1977)**									
No	1064	21 (2.0)	1	-	-	77 (7.2)	1	-	-
Yes	913	36 (3.9)	2.04	1.18–3.52	0.013	59 (6.5)	0.89	0.62–1.26	0.556
**Container washing (n = 1977)**									
No	1489	48 (3.2)	1	-	-	120 (8.1)	1	-	-
Yes	488	9 (1.8)	0.56	0.28–1.16	0.154	16 (3.3)	0.39	0.23–0.66	<0.001

(*)Fisher´s exact test.

#### Multivariate analysis

The final multivariate model of risk factors independently associated with living in a hot spot household is shown in Table 5. Being a domestic worker/housewife, living in a shack, storing water in containers, have litter outdoors and using repellent were all positively associated with living in a hot spot household. The positive association with the use of mosquito repellent could be related to a perceived higher amount of mosquitoes in their place of residence. Individuals living in dwellings with more than six people were negatively associated with dengue hot spot households. Table 6 displays the final model of factors independently associated with living within a hot spot block. The model shows that the occupation of domestic worker/housewife, the number of persons per household, household crowding and having had screened windows/doors were all positively associated with living in a hot spot block. Individuals that lived in a household with more than 5 rooms (bigger houses), who used insecticide, and washed their water containers, were negatively associated with living in a hot spot block.

**Table 5:** Multivariate logistic regression model of risk factors associated with living in a hot spot household.

Final model of risk factors for dengue hot spots at household level (n = 1983)			
	OR	CI_95_	*P*-value
**Domestic worker/housewife**			
No	1	-	-
Yes	2.86	1.45–5.64	0.002
**Type of housing**			
House/Apartment/ other	1	-	-
Rancho^a^	13.55	5.40–34.02	<0.001
**Number of persons per household**			
1–6	1	-	-
≥7	0.42	0.24–0.76	0.004
**Water storage in containers**			
No	1	-	-
Yes	1.95	1.09–3.52	0.024
**Litter outdoors**			
No	1		
Yes	2.37	1.38–4.08	0.002
**Use of repellent**			
No	1	-	-
Yes	2.03	1.17–3.51	0.011

^a^In Venezuela, the word “rancho” is used to define a “shack”, an informal substandard type of housing typical of slum areas.

**Table 6:** Multivariate logistic regression model of risk factors associated with living in a hot spot block.

Final model of risk factors for hot spots at block level (n = 1983)			
	OR	CI_95_	*P*-value
**Domestic worker/housewife**			
No	1	-	-
Yes	2.01	1.17–3.46	0.012
**Number of household rooms**			
1–4	1	-	-
≥5	0.88	0.82–0.95	<0.001
**Number of persons per household**			
1–6	1	-	-
≥7	1.98	1.40–2.80	<0.001
**Crowding (number of people/room)**			
0.17–1.49	1	-	-
1.5–8.0	2.34	1.66–3.31	<0.001
**Use of insecticide**			
No	1	-	-
Yes	0.43	0.30–0.63	<0.001
**Container, Tanks Washing**			
No	1	-	-
Yes	0.39	0.23–0.66	<0.001
**Screened Windows/Doors**			
No	1	-	-
Yes	2.28	1.10–4.74	0.026

## Discussion

To our knowledge, this is the first study to apply spatial analysis techniques in Venezuela to determine areas of higher transmission of dengue coupled with the identification of risk factors that may explain the higher endemicity within these clusters. These methods are increasingly being used to understand dengue epidemiology with few studies published so far in this area of research in the Americas [31,32,33,34,35].

We detected spatial clusters of dengue seroprevalence and identified the risk factors for dengue transmission associated with these clusters in a dengue hyperendemic city in Venezuela. Hot spots of recent dengue infection at household and block level occurred in all 3 neighborhoods under study. However, one neighborhood, Caña de Azucar, concentrated the majority of hot spots and accounted for most dengue transmission across the whole studied area showing the focal nature of this mosquito-borne viral infection. Indeed, our results indicated that most of the clustering distance did not extend beyond 100 m suggesting that an underlying spatial process of dengue transmission is acting at such small scale. Conditions that enhanced the risk of transmission and infection by dengue virus in a hot spot household or a hot spot block were related with occupation (being a domestic worker/housewife), lower socio-economic status (to live in a shack, crowded conditions, more people per room), the creation of potential mosquito breeding sites (to store water in containers and having litter outdoors) and mosquito preventive measures (have screened windows/doors, the usage of insecticide or repellent, and container washing).

Spatial analysis techniques applied to vector borne diseases have proven useful to define high risk areas of transmission and factors associated with this risk, while informing health authorities on better targeted control actions as well as generating models applicable to other regions [15,36,37,38]. Using seroprevalence data from three neighborhoods in Maracay city [14] we drew risk maps and determined hot spots of dengue transmission at household and block level. An important proportion of dengue infections are clinically inapparent [39,40] contributing to increased viral transmission. Therefore, the use of seroprevalence data over incidence (symptomatic cases) may give a more accurate estimate of high dengue transmission areas. Risk maps of past dengue seroprevalence showed a certain spatial homogeneity as the majority of blocks had a past seroprevalence > 40% following the high overall prevalence found in the population under study (77.4%) [14] (Figs 3a and 4a). This also resulted in the absence of identifiable hot spots for past dengue at any spatial level. The neighborhoods included in our study have been reported as areas of perennial dengue transmission, tending to maintain an infectious cycle of dengue outside of the rainy season [15,41]. The progressive entry of the different serotypes of dengue in Venezuela has been associated with the major epidemics in the country [14,41,42,43,44]. In 2010, our fieldwork coincided with one of the major epidemics of dengue in Venezuela [19] probably resulting in the observed high seroprevalence. Similar observations of dengue post-epidemic prevalence were observed in American Samoa in 2010 with a seroprevalence of 95.6% [45].

Spatial heterogeneity was revealed when drawing risk maps of recent dengue seroprevalence. Recent dengue infections were recorded in 10% (n = 200) of the individuals under study [14]. Interestingly, areas with high recent seroprevalence coincided with areas of increased past dengue seroprevalence which could indicate the persistence of dengue transmission in these locations. A total of 39 significant hot spots for recent dengue at household level and 9 significant hot spots at block level were found, most of them in Caña de Azucar neighborhood, which confirms the higher risk of recent dengue transmission in this neighborhood (Fig 3b). Caña de Azúcar neighborhood is one of the most densely populated areas of Maracay and living in this neighborhood was associated to a lower socio-economic status and a higher proportion of potential breeding sites [14]. Individuals living in this neighborhood were significantly more prone to store water at home, live in smaller houses and in more crowded conditions than people residing in the other two neighborhoods [14], This findings suggest an increased chance of dengue transmission in this area [12, 31].

The transmission of dengue in our area of study was highly focal (radius = 20–110 meters) suggesting that at domestic level the necessary conditions for oviposition, growth, feeding and reproduction of the mosquito vector exist. In agreement with our findings, other studies have reported a short flight range for *Ae*. *aegypti* (radius <40 m) where the vector tends to be spatially clustered at household level in relation to the occurrence of indoor breeding sites [46] and abundant human hosts [47]. Other authors found that female mosquitoes do not visit more than 3 houses in a lifetime [48]. In Iquitos, Peru, researchers registered a mosquito flight range of 10–30 meters, and concluded that in this area the vector does not fly away from the water containers where they breed [49]. Likewise, it has been reported that dengue cases cluster within households [50]. The small spatial dependency scale (20–110 meters) and the finding that people who spend more time indoors such as domestic workers/housewives were at a higher risk of recent dengue (Tables 5 & 6) compared to those who had jobs away from home strongly indicates that transmission occurs mainly at home as suggested in other studies [14,51,52]. Here, it is important to point out that our study is focused mainly on the local spatial scale which is much related to the short-range flight of mosquito dispersal; however, we also highlight the relevance of the human movement to the spatial dynamic of dengue at large spatial scale. It is the interaction between infected mosquito dispersal at very small scale and infected human movements at large scale that underlie the dynamics of dengue transmission through space and time.

Proxy markers of poverty or lower socio-economic status were strongly associated with hot spot households and blocks and were more relevant spatially than those found in a previous study with classical analysis of seroprevalence data [14]. People living in a shack were associated with living in a hot spot household, while those in smaller households (<5 rooms) and in crowded conditions were related to inhabiting a hot spot block (Tables 5 & 6). In our studied neighborhoods, population growth and the need for extra income resulted in the sub-division of one-family dwellings into smaller “apartments” to house two or more families. The consequences are more crammed living conditions and deprivation [53]. Studies in Brazil [32] and Ecuador [54] determined that dengue clusters or hot spots are mainly located in poor areas. Unplanned urbanization and precarious living circumstances are characterized by the lack of proper public services (piped water supply, electricity, garbage collection, sewage) and crowding favoring the transmission of dengue [14,34,55]. Other authors found that increased dengue transmission was associated with socio-economic factors rather than climatic factors [56].

We explored the mosquito preventive measures taken by the population under study in order to understand if they had any effect in reducing dengue transmission. While some measures showed a protective effect, i.e., individuals that used insecticide sprays and regularly washed their water containers were less prone to inhabit a hot spot block, other actions showed an opposite result. People that lived in households with screened windows/doors and that used mosquito repellent had a higher probability of residing in a hot spot block or household, respectively. Although this may be difficult to explain, we hypothesize that the latter results may be indirect markers of higher mosquito density prompting individuals to implement these protective measures, as reported by other authors [57].

Potential breeding sites were only found independently associated with hot spots at household but not at block level in multivariate analysis. The presence of litter in household premises and storing water in containers enhanced the odds of living in a hot spot household at least twice (Table 5). Several studies have linked the presence of cans, small canisters or other types of containers accumulated in and around household grounds as potential breeding sites that may be extending the possibilities of oviposition of dengue vector mosquitoes beyond the rainy season [36,58,59]. Poor piped water supply involves storing water in diverse type of containers creating a suitable environment for the growth and development of *Aedes spp*. especially during the dry season, consequently keeping a perennial dengue transmission in the population [15,47,60,61]. Storing water in containers in Venezuela is a practice that has been reported for several years [36,60]. The neighborhoods of our study have been subjected to long-lasting deficits in public services, especially prolonged interruptions of piped water supply and electricity. This has prompted the population to maintain water storage at home all year round. The worsening socio-economic situation in Venezuela can predict the perennial maintenance of dengue transmission and an increase in the frequency of epidemics as seen recently [19]. Targeting the identified hot spot areas with strategies such as source reduction and community education [49] may result in a cost-effective manner to improve dengue control in Venezuela and similar endemic areas. A limitation of our work is the absence of entomological data that may complement our results and provide more clues about the focal transmission of dengue in these urban areas. However, our results can be supported by the identified risk factors for dengue infection performed in the same population in a previous study [14].

## Conclusions

The application of geographic information systems and spatial analysis for the detection of areas of greater transmission of DENV is of vital importance for the prevention and control of vector-borne diseases such as dengue. We used seroprevalence data to understand the spatial spread and clustering of dengue in a hyperendemic city in Venezuela. In contrast with incidence data, seroprevalence has the advantage to include inapparent as well as symptomatic infections giving a more realistic view of the high transmission risk areas. Moreover, we performed an analysis of risk factors at a fine spatial scale comparing individuals living within hot spots at household and block level versus those that lived outside these geographical areas. We determined that most hot spots clustered in one neighborhood and that transmission occurs at a very small scale (radius of 20–110 m) and domestically giving the possibility to target the scarce resources to this specific area. Secondly, poverty-related factors and those related to potential breeding sites were associated with these hot spots pointing to measures that can and should be taken promptly as reported previously [15]. However, political will and channeling enough resources to alleviate low socio-economic conditions and improve public services are essential for success in dengue control.

The identification of hot spots of dengue transmission and the factors associated with these clusters are important tools to inform health authorities to improve and target control measures against dengue. Further studies are needed to define if these hot spot areas are maintained through time while similar studies can be applied to other vector-borne infections such as malaria, chikungunya and Zika virus affecting both Venezuela and other Latin American countries.

## Supporting Information

S1 Checklist STROBE Checklist. (DOC)

S1 Dataset DS_S1 Codebook GIS Baseline databaseDS_S1 GIS Baseline databaseDS_S1 Dengue prevalence per block Candelaria_Cana AzucarDS_S1 Dengue Prevalence per block_Cooperativa
(ZIP)
