# Supplementary material for: Spatial Analysis of Dengue Seroprevalence and Modeling of Transmission Risk Factors in a Dengue Hyperendemic City of Venezuela
Source: PLoS Negl Trop Dis. 2017 Jan 23;11(1):e0005317. doi: 10.1371/journal.pntd.0005317 (PMC5289626; doi:10.1371/journal.pntd.0005317)
Supplement: S1 Dataset — DS_S1 Codebook GIS Baseline databaseDS_S1 GIS Baseline databaseDS_S1 Dengue prevalence per block Candelaria_Cana AzucarDS_S1 Dengue Prevalence per block_Cooperativa (ZIP) [file pntd.0005317.s002.zip › DS_S1 Codebook GIS Baseline database.doc]

**Dengue Project in Venezuela**

**CODEBOOK**

| **Variable name** | **Meaning** | **Values** |
| --- | --- | --- |
| **id** | Individual unique identifier | 0001 to 2012 |
| **Household code** | Unique identifier of each studied household | Number VIV-0001 to VIV-840 |
| **gender** | Gender: Male and Female | M= 0; F= 1 |
| **No work no study** | Person does not study nor work | No work no study=1  Other=0 |
| **study** | Are you studing? | Yes= 1; No= 0; |
| **work** | Do you work? | Yes= 1; No= 0; |
| **pubserv** | Do you have all public services?  Pipped water, electricity, garbage collection, sewage) | Yes= 1; No= 0 |
| **water_stor** | General water storage at home | Yes= 1; No= 0 |
| **tank_stor** | Water storage in tanks | Yes= 1; No= 0 |
| **container_stor** | Water storage in containers | Yes= 1; No= 0 |
| **litter** | Litter outdoors | Yes= 1; No= 0 |
| **tires** | Used car tires outdoors | Yes= 1; No= 0 |
| **bottles** | Bottles outdoors | Yes= 1; No= 0 |
| **flower_vases** | Indoor flower vases | Yes= 1; No= 0 |
| **screened_w** | Screened Windows/doors | Yes= 1; No= 0 |
| **mosqui_net** | Mosquito net | Yes= 1; No= 0 |
| **insect** | Insecticide | Yes= 1; No= 0 |
| **repe** | Repellent | Yes= 1; No= 0 |
| **container_wash** | Daily washing of containers and tanks | Yes= 1; No= 0 |
| **occupation_cat** | Categories of occupation | 0=Unemployed  1=Student  2=Housewife/ Domestic worker  3=Manual worker  4=Merchant/ Employee/ Office worker  5=Professional/ University staff |
| **age_cat** | Categories of age | 5-10=10  11-15=15  16-20=20  21-30=30 |
| **Domes_DIC** | Domestic worker | 0=all the rest  1=domestic worker |
| **shack_DIC** | Categorical of type of house | 0= house, apartment, other  1= schack |
| **dur_res** | Duration of residence divided in two categories | Quartiles 1-3 (0-16years)= 0  Quartile 4 (≥17 yrs) = 4 |
| **House_cluster** | Hot spots at household level. | 0= not hotspot household  1=hotspot household |
| **Blocks_cluster** | Households that are within a hot spot block | 0=no household within a hotspot block  1= household within a hotspot block |
| **block_code** | Unique identifier of blocks | Number: ## |
| **persons_house** | Number of persons per household | 0=1-6  1= ≥7 |
| **crowding** | Crowding (people/room) | 0.17-1.49=0  1.5-8=1 |
| **rooms_cat** | Number of household rooms | ≤4hab =4 ; ≥5 hab=5 |
